# Peripheral blood monocyte status is a predictor for judging occurrence and development on sepsis in older adult population: a case control study

**DOI:** 10.1186/s12873-023-00779-w

**Published:** 2023-01-31

**Authors:** Qian Gao, Li Yang, Fei Teng, Shu‑Bin Guo

**Affiliations:** 1 Emergency Department, Beijing Shijitan Hospital, Capital Medical University, No. 10 Tieyi Road, Yangfangdian, Haidian District, Beijing, 100038 China; 2 Emergency Medicine Clinical Research Center, Beijing Key Laboratory of Cardiopulmonary Cerebral Resuscitation, Beijing Chao-Yang Hospital, Capital Medical University, No. 8, South Road of Worker’s Stadium, Chaoyang District, Beijing, 100020 China

**Keywords:** Sepsis, Monocyte subsets, Cytokine, Aging

## Abstract

**Background:**

Peripheral blood monocytes are important immune modulatory cells that change during aging. Previous studies on sepsis and monocytes did not distinguish between age groups, especially in the older adult population. The mechanisms of monocyte subsets and function are not well-understood in the aging context with sepsis.

**Methods:**

Monocyte subsets were measured using flow cytometry in 80 sepsis patients and 40 healthy controls. Plasma cytokine levels were measured using cytokine antibody arrays.

**Results:**

The percentage of MO3 (CD14 + CD16 + +)/monocytes was higher in sepsis patients than in controls (*P* = 0.011), whereas the percentage of MO1 (CD14 +  + CD16 −)/monocytes was higher in septic shock patients and 28-day death group than in those without shock and 28-day survival group (*P* = 0.034, 0.038). Logistic regression analysis showed that the percentage of MO3/monocytes (OR = 1.120, *P* = 0.046) and plasma level of monocyte chemoattractant protein (MCP)-1 (OR = 1.006, *P* = 0.023) were independently associated with the occurrence of sepsis, whereas the percentage of MO1/monocytes (OR = 1.255, *P* = 0.048) was independently associated with septic shock. The receiver operating characteristic (ROC) curve showed that the area under the curve (AUC) of MO3/monocyte percentage in combination with MCP-1 plasma level (AUC = 0.799) for predicting sepsis was higher than that of each parameter alone (*P* < 0.001). The AUC of MO1/monocyte percentage with the value 0.706 (*P* = 0.003) was lower than the AUC of SOFA (sequential organ failure assessment) score with the value 0.966 (*P* < 0.001) for predicting septic shock, but the value of the two AUCs were similar for predicting 28-day mortality (AUC = 0.705, 0.827; *P* = 0.020, *P* < 0.001). The AUC of MO1/monocytes percentage in combination with SOFA score for predicting 28-day mortality was higher than that of each parameter alone (AUC = 0.867, *P* < 0.001). Using a cut-off of 58.5% (for MO1/monocytes determined by ROC) could discriminate between survivors and non-survivors on Kaplan–Meier curves for 28-day mortality with a positive predictive value of 77.4%.

**Conclusion:**

The MO3/monocyte percentage and plasma MCP-1 level were independent predictors of sepsis occurrence, whereas the percentage of MO1/monocytes was an independent predictor of prognosis in the Chinese Han older adult population.

**Trial registration:**

Registration number: ChiCTR2200061490, date of registration: 2022–6-26 (retrospectively registered).

## Background

Sepsis is a severe infection with a series organ dysfunction that involved a complicated progress between pro- and anti-inflammatory course. Peripheral blood monocytes are critical immune cells that play important roles in immune responses. Human monocytes show different functional based on CD14 (lipopolysaccharide receptor) and CD16 (FcγIII receptor) expression on their cell surface [1, 2]. According to the expression of this two receptors, monocytes can be classified into “classical monocytes” with strong expression of CD14 and negative expression of CD16 (CD14 +  + CD16 −), “intermediate monocytes” with both expression CD14 and CD16 (CD14 +  + CD16 +), and “nonclassical monocytes” with mainly expressing CD16 (CD14 + CD16 + +) [1].

Aging causes changes in the immune system and represents a critical healthcare concern. It is characterized chronic low-grade inflammation in the older adult population including increased cell senescence and altered circulating level of cytokines [3, 4]. Monocytes are dynamic immune modulatory cells changing with aging. Previous studies on sepsis and monocytes did not distinguish age context mostly, especially in the older adult population. The mechanisms regulating monocyte phenotype and function are not well-understood in the aging context. Therefore, we investigated the association of different peripheral blood monocyte subsets and their secreted cytokines on the occurrence and development of sepsis in the older adult Chinese Han population.

## Methods

### Patients and control subjects

The participants were patients from two hospitals who were admitted to the emergency department (ED). The patients included were diagnosed with sepsis defined by the 2016 International Diagnostic Criteria for Sepsis 3.0 as life-threatening organ dysfunction caused by a dysregulated host response to infection and satisfied the age above 65. For clinical operationalization, organ dysfunction is indicated by an increase in the Sequential Organ Failure Assessment (SOFA) score by ≥ 2 points. Patients with septic shock are clinically identified by a vasopressor requirement to maintain a mean arterial pressure of ≥ 65 mmHg and plasma lactate level of > 2 mmol/L in the absence of hypovolemia [5]. The exclusion criteria were: (a) congenital and/or acquired immunodeficiency diseases, (b) long-term use of corticosteroids or immunosuppressive drugs, (c) patients with HIV infection or cancer, (d) death within 2 days of the onset of sepsis, signs of sepsis occurring more than 3 days prior to admission, (e) declined to participate. Blood samples were collected within 24 h after the sepsis criteria were met. A healthy control group was also from the two hospitals who were admitted to the physical examination centres. Subjects were excluded if they had hypertension, diabetes, coronary heart disease, or other serious diseases of the brain, lung, liver, or kidney. Blood samples were collected on the same day as admission to the physical examination centre.

### Data collection

The clinical characteristics of patients, including age, sex, and laboratory examination results, were recorded after the onset of sepsis. The SOFA score and SAPS II score (simplified acute physiology score) were calculated based on related clinical and demographic data. The following outcome of survival condition (survival or death) was collected after 28 days during follow-up.

### Flow cytometry

Peripheral whole blood was collected into ethylenediaminetetraacetic acid (EDTA) anticoagulant tubes. The antibodies were purchased from BD Biosciences (San Jose, CA, USA). Erythrocytes were lysed and stained by a technician who was blinded to the study. Cells were stained in the dark on ice for 30 min and washed twice. At least 10,000 monocytes were acquired using a BD FACS Aria II flow cytometer (BD Biosciences). Peripheral whole-blood cell analysis was performed using antibodies specific for human CD45 (clone HI30), CD14 (clone M5E2), and CD16 (clone 3G8). All antibodies were previously titred and optimized, depending on the fluorophore used. Forward scatter and side scatter and CD14 and CD16 positive signals based on isotype-matched control staining were used to gate monocytes. The forward scatter area vs. forward scatter height was used to gate single cells. The analysis was performed using FlowJo software (v. 10.0.8; Tree Star, Ashland, OR, USA). The results were expressed as percentages.

### Cytokine testing

Peripheral venous blood samples were collected in tubes containing potassium EDTA and immediately centrifuged at 3000 × g for 10 min at ambient temperature. Plasma from the supernatant was extracted and frozen at − 80 °C until analysis. Cytokines were tested using cytokine antibody arrays (Quantibody® Human Inflammation Array 1) containing 10 human cytokines (interferon [IFN]-γ, interleukin [IL]-1α, IL-1β, IL-4, IL-6, IL-8, IL-10, IL-13, monocyte chemoattractant protein-1 [MCP-1], and tumour necrosis factor [TNF]-α). The fluorescence signal values were used for semiquantitative evaluation.

### Statistical analysis

Normally and non-normally distributed data were described as mean ± standard deviation and median (interquartile range) respectively. Independent sample t-tests, Mann–Whitney U-tests and chi-square tests were used to compared the differences between groups as appropriate. Binary logistic regression was used to identify variables associated with the occurrence of sepsis, septic shock and 28-day mortality. The area under the curve (AUC) of receiver operating characteristic (ROC) curves was used to compare the prediction of sepsis occurrence, septic shock, and 28-day mortality in sepsis. Using cut-off values determined by ROC curves, comparisons of survival distributions were assessed by the log-rank test from Kaplan–Meier survival curves. All statistical tests were two-tailed, and statistical significance was set at *P* < 0.05. All data were analysed using SPSS 23.0 software.

## Results

### Patient characteristics

**Fig. 1:**
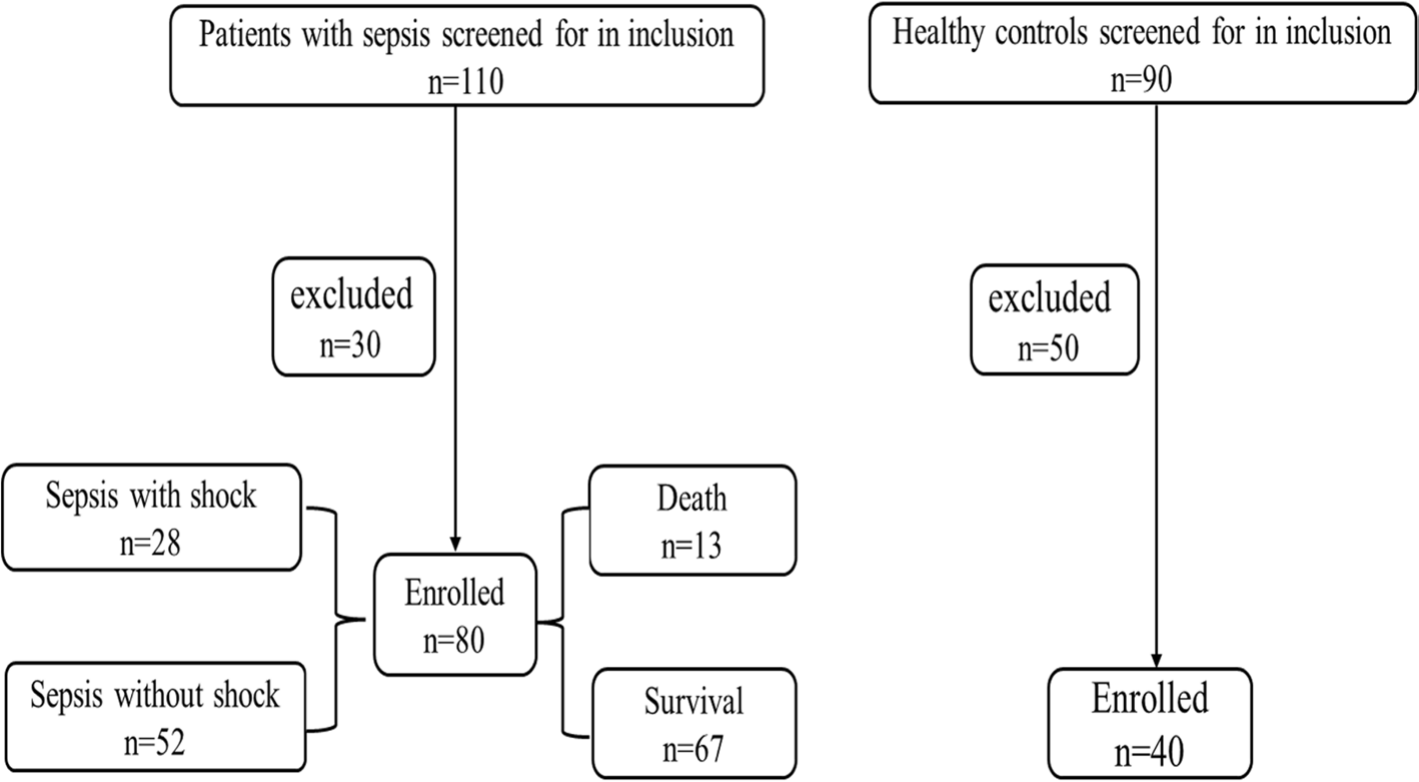
Flow diagram of included patients and controls with reasons for exclusion

A total of 80 older adults Chinese Han sepsis patients and 40 healthy controls matched for sex, age, and race were included in this study (Fig. 1). The patients were divided into septic shock group (28) and septic group (patients without shock) (52) according to disease severity, death group (13) and survival group (67) according to the 28-day mortality. The demographic and clinical characteristics of patients are presented in Table 1. Septic shock group had higher SOFA and SAPS II scores than septic group. Patients in the septic shock group had higher 28-day mortality than those without shock group. Other parameters such as PCT, CRP and ESR shown no significant difference between the groups.

**Table 1:** Baseline characteristics of all participants

	All patients		Controls	*P* value
	Septic shock group	Septic group		
	*N* = 28	*N* = 52	*N* = 40	
Age (yrs)	80.0 ± 8.4	81.1 ± 8.6	76 ± 5.8	0.085
Male, n (%)	17(60.7)	26(50)	24(40)	0.530
PCT (ng/ml)	18.8 ± 40.4	11.5 ± 23.0		0.476
CRP (mg/L)	140.3 ± 94.5	110.6 ± 79.5	/	0.194
ESR (mm/h)	50.0 ± 33.8	51.4 ± 31.9	/	0.935
SOFA score	11.1 ± 1.4	5.3 ± 2.4	/	** < 0.001**
SAPS II	45.5 ± 10.5	37.6 ± 8.9	/	**0.001**
28-day mortality, n (%)	10(35.7)	3(5.8)	/	**0.003**

Data are described by mean ± SD, median (Q1, Q3), number (%)

*PCT* Procalcitonin, *CRP C* Reactive protein, *ESR* Erythrocyte sedimentation rate, *SOFA* Sequential organ failure assessment, *SAPS II* Simplified acute physiology score II

*P* < 0.05 was indicated in bold

### Monocyte subsets between patients and controls

**Fig. 2:**
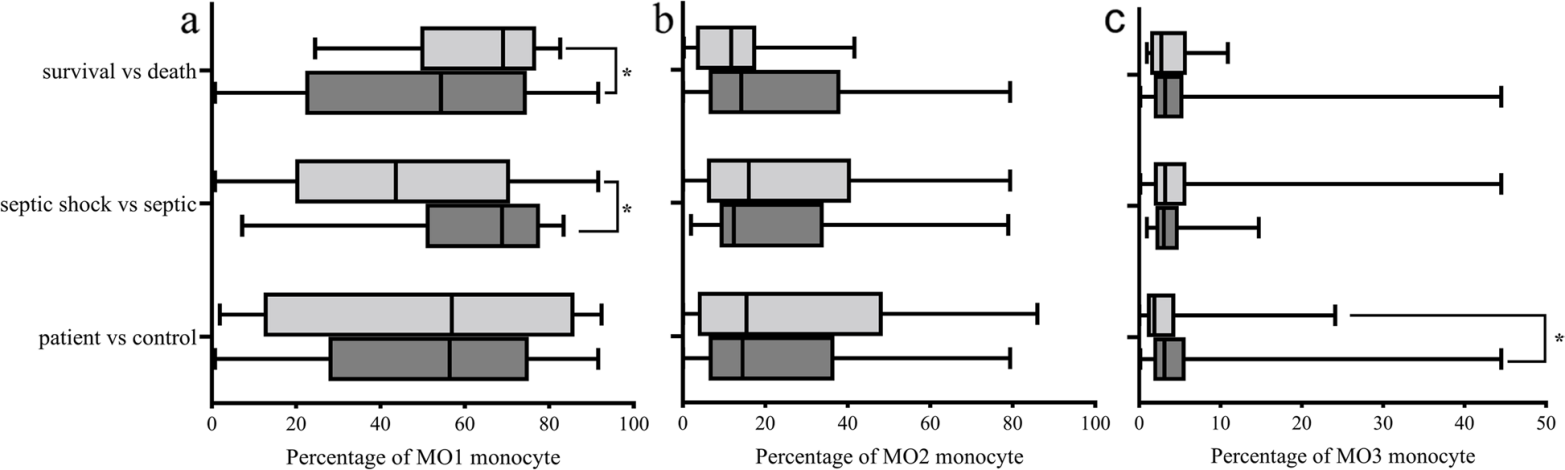
Comparing the subsets of peripheral blood monocytes between groups [patient and control, septic shock and septic patients (no shock), patients in 28-day survival and death]. Figure **a**, **b**, **c** represent the percentage of MO1 (CD14 +  + CD16-), MO2 (CD14 + CD16 +), MO3 (CD14 + CD16 + +) monocyte in the three groups respectively, *p = 0.011, 0.034, 0.038

We defined CD14 +  + CD16 − (classical) monocyte as MO1 monocyte, CD14 +  + CD16 + (intermediate) monocyte as MO2 monocyte, CD14 + CD16 +  + (non-classical) monocyte as MO3 monocyte. Comparisons among the groups of percentage of monocyte subsets were illustrated in Fig. 2. The percentage of MO3 monocytes was higher in patients than in controls [3.1% (1.7%, 5.7%) vs. 1.9% (0.9%, 4.4%), *P* = 0.011] (Fig. 2c), whereas percentages of MO1 monocytes and MO2 monocytes shown no differences between patients and controls [56.4% (28.0%, 75.1%) vs. 56.9% (12.3%, 86.0%), *P* = 0.602; 14.5% (6.3%, 36.8%) vs. 15.5 (3.7%, 48.5%), *P* = 0.597, respectively] (Fig. 2a, b). Further analysis revealed a high percentage of MO1 monocytes in septic shock patients and 28-day death group than in those without shock group and 28-day survival group[68.8% (50.7%, 77.8%) vs. 40.8% (19.7%, 70.7%), *P* = 0.034; 54.3% (22.2%, 74.7%) vs. 70.9% (49.4%, 76.9%), *P* = 0.038] (Fig. 2a), no differences were observed on percentage of MO2 monocytes and MO3 monocytes between these groups [12.4% (8.9%, 34.2%) vs. 14.8% (6.8%, 41.6%), *P* = 0.988; 3.1% (2.0%, 4.9%) vs. 3.0% (1.7%, 5.9%), *P* = 0.992, respectively] (Fig. 2b, c). The percentages of MO2 and MO3 monocyte showed no significant differences between survival and death group according to 28-day mortality [14.2% (6.3%, 38.2%) vs. 15.2% (6.1%, 23.7%), *P* = 0.588; 3.2% (1.79%, 5.43%) vs. 2.7% (1.4%, 5.9%), *P* = 0.616, respectively] (Fig. 2b, c).

### Differences in cytokines secreted by monocytes between patients and controls

**Fig. 3:**
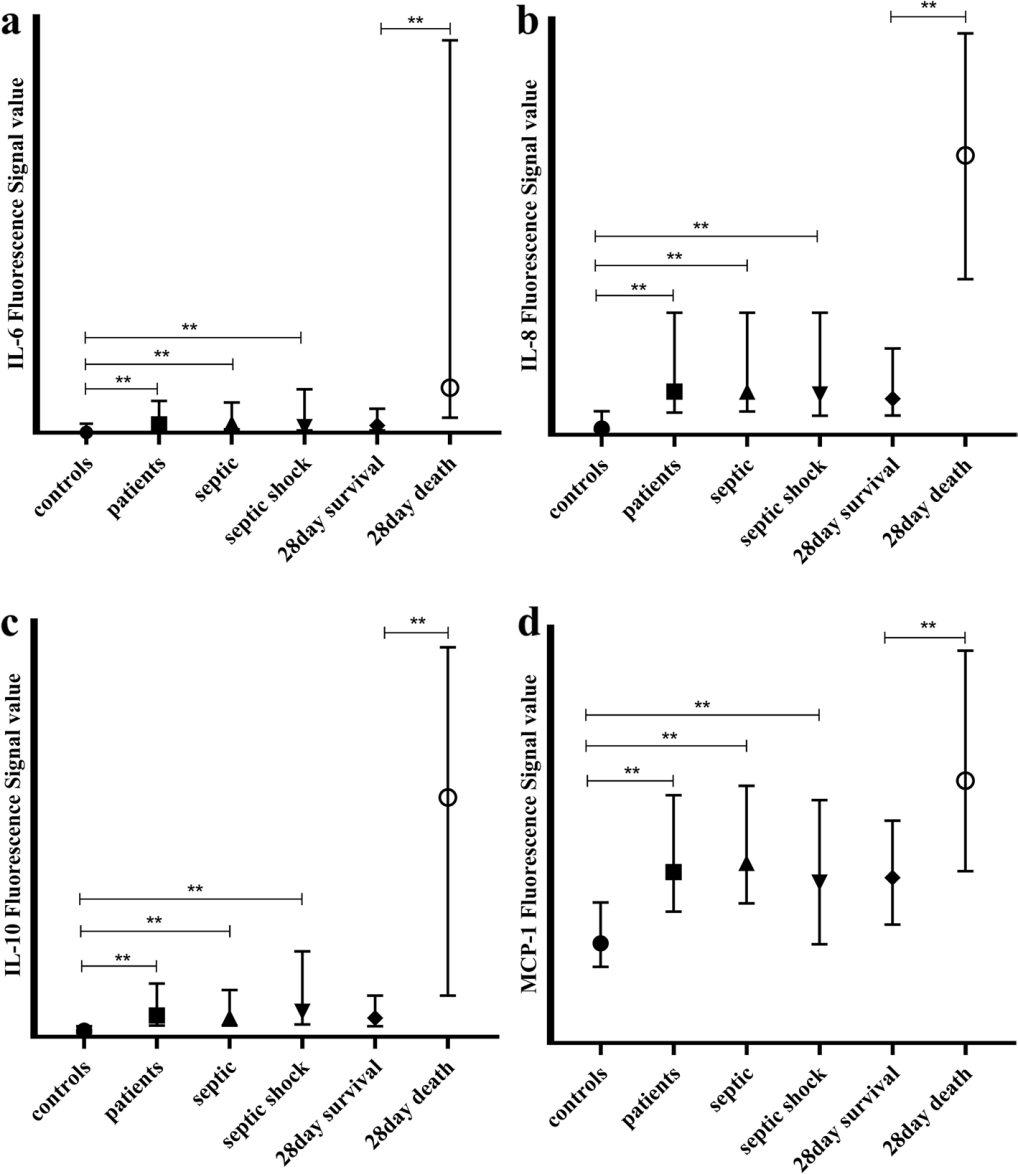
Illustrated the plasma levels of IL-6, IL-8, IL-10 and MCP-1 (Fig. **a**, **b**, **c**, **d** represented IL-6, IL-8, IL-10, MCP-1 respectively) between groups (patients vs controls, septic vs controls, septic shock vs controls, septic shock vs septic, 28-day survival vs death). ** all *p* < 0.001

Monocytes mainly secrete IL-6, IL-8, IL-10, MCP-1, TNF-α, and IL-1β [6]. In addition, we selected other important inflammatory cytokines, such as IL-1α, IL-4, IL-13, and IFN-γ, for detection. We found that the plasma levels of IL-6 (Fig. 3a), IL-8 (Fig. 3b), IL-10 (Fig. 3c), and MCP-1 (Fig. 3d) were upregulated in all patients (all *P* value < 0.001), the septic group (all *P* value < 0.001), and the septic shock group (all *P* value < 0.001) compared with those in control groups. However, no differences were observed between patients with septic shock and those without shock. Plasma levels of IL-6, IL-8, IL-10, and MCP-1 were also upregulated in non-survivors compared with those in survivors according to the 28-day mortality (all *P* value < 0.001). The other cytokines mentioned above were not significantly different among these groups, although slight differences were observed.

### MO3 monocyte percentage and MCP-1 level as independent predictors of the occurrence of sepsis disease

**Fig. 4:**
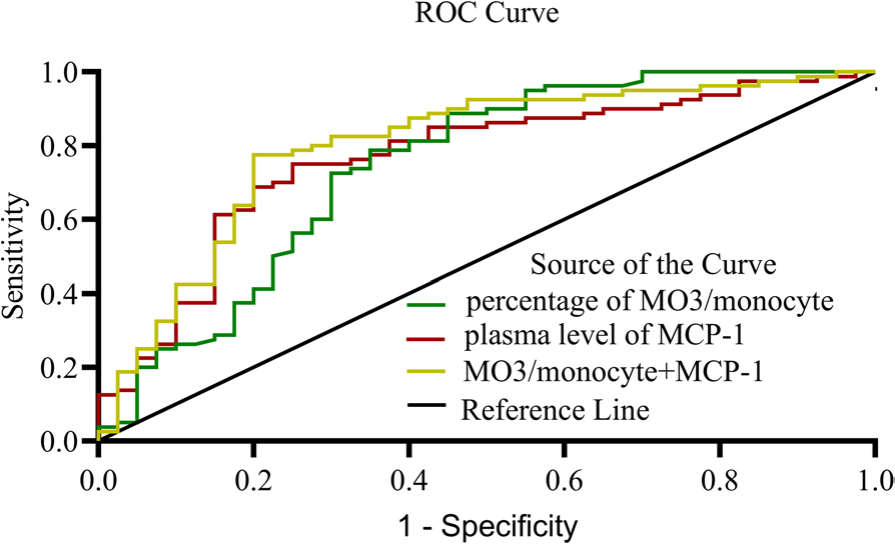
Receive operating characteristic (ROC) curve for predicting sepsis. AUCs: the percentage of MO3/monocytes (green line), 0.745; plasma level of MCP-1 (red line), 0.765; percentage of MO3/monocytes in combination of plasms level of MCP-1(yellow line), 0.799

In the subsequent multivariate logistic regression analysis, we found that MO3/monocytes (β = 1.113, OR = 1.120, CI: 1.002, 1.251, *P* = 0.046) and the MCP-1 plasma level (β = 0.006, OR = 1.006, CI: 1.001, 1.010, *P* = 0.023) were independently associated with the occurrence of sepsis disease (Table 2). The ROC curve showed that the AUCs of the MO3/monocyte percentage and MCP-1 plasma level for predicting sepsis were 0.745 and 0.765 (*P* < 0.001, *P* < 0.001), respectively. Moreover, the AUC of 0.799 (*P* < 0.001) for the percentage of MO3/monocytes in combination with the plasma level of MCP-1 for predicting sepsis was significantly higher than that for each parameter alone. The detailed data are presented in Table 3 and Fig. 4.

**Table 2:** Logistic regression analysis of independent factors for occurrence of sepsis disease

variable	β	SE	Wald	*P* value	Odds ratio	95% confidence interval for EXP(B)	
						Lower limit	Upper limit
MO3/monocyte	1.113	0.057	3.868	**0.046**	1.120	1.002	1.251
IL-6	0.000	0.002	0.030	0.862	1.000	0.997	1.004
IL-8	0.058	0.038	2.274	0.132	1.059	0.983	1.142
IL-10	-0.008	0.008	1.053	0.305	0.992	0.978	1.007
MCP-1	0.006	0.002	6.122	**0.023**	1.006	1.001	1.010
Constant	-1.446	0.907	2.543	0.047	0.164		

MO3 CD14 + CD16 +  + monocyte

*IL* Interleukin, *MCP* Monocyte chemoattractant protein

**Table 3:** Area under the curve of parameters for predicting sepsis in all candidates

Variable	AUC	*P* value	95% Confidence interval	
			Lower limit	Upper limit
Percentage of MO3/monocyte	0.745	< 0.001	0.710	0.889
Plasma level of MCP-1	0.765	< 0.001	0.673	0.857
Combination of MO3/monocyte with MCP-1	0.799	< 0.001	0.643	0.848

MO3 CD14 + CD16 +  + monocyte

*MCP* Monocyte chemoattractant protein

### Percentage of MO1 monocytes as a new independent predictor of sepsis severity and prognosis

**Fig. 5:**
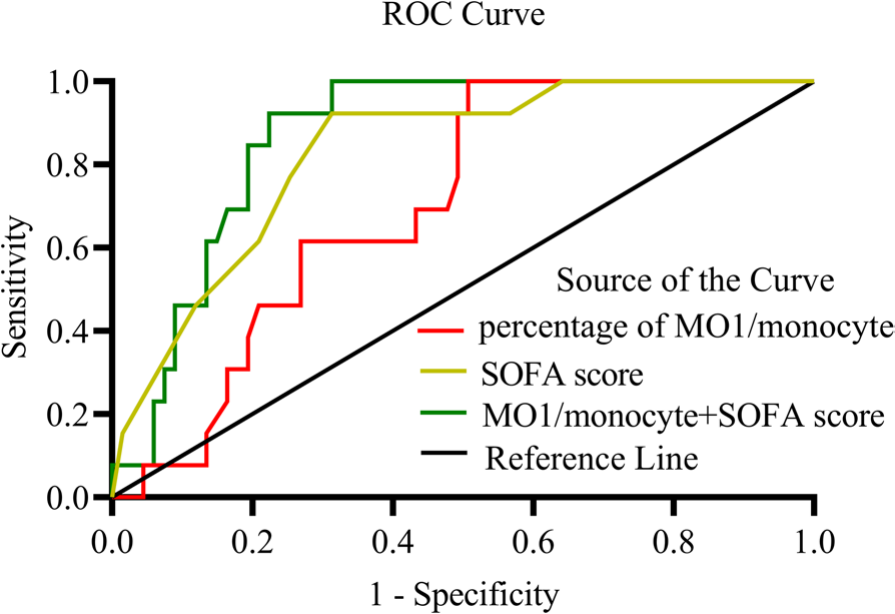
Receive operating characteristic (ROC) curve for predicting 28-day mortality in all sepsis patients. AUCs: the percentage of MO1/monocytes (red line), 0.705; SOFA score (yellow line), 0.827; the percentage of MO1/monocytes in combination with SOFA score (green line), 0.867

The MO1/monocyte percentage (β = 0.227, OR = 1.255, CI: 1.002, 1.572, *P* = 0.048) and SOFA score (β = 1.951, OR = 7.036, CI: 1.529, 32.383, *P* = 0.012) were independently associated with septic shock according to the disease severity using logistic regression analysis (Table 4). The ROC curve showed that the AUC of the percentage of MO1/monocytes was 0.705 (*P* = 0.003), but was lower than that of the SOFA score and SASP II for predicting septic shock in all patients (AUC = 0.966, 0.737; *P* < 0.001, = 0.001 respectively). Interestingly, the AUC of the percentage of MO1/monocytes was similar to that of the SOFA score for predicting 28-day mortality in all patients (Table 5), and the prognostic value of MO1/monocytes in combination with the SOFA score for predicting 28-day mortality was significantly higher than that for each parameter alone (Table 5 and Fig. 5).

**Table 4:** Logistic regression analysis of independent factors for septic shock in all patients

variable	β	SE	Wald	*P* value	Odds ratio	95% confidence interval for EXP(B)	
						Lower limit	Upper limit
MO1/monocyte	0.227	0.115	3.906	**0.048**	1.255	1.002	1.572
SOFA score	1.951	0.779	6.275	**0.012**	7.036	1.529	32.383
SASPII	-0.060	0.066	0.837	0.360	0.942	0.828	1.071
Constant	-37.508	16.174	5.378	0.020	0.000		

MO1 CD14 +  + CD16 − monocyte; MO2 CD14 +  + CD16 + monocyte; MO3 CD14 + CD16 +  + monocyte

*SOFA* Sequential organ failure assessment, *SASPII* Simplified acute physiology score II

**Table 5:** Area under the curve of parameters for predicting 28-day mortality in all patients

Variable	AUC	*P* value	95% Confidence interval	
			Lower limit	Upper limit
Percentage of MO1/monocyte	0.705	0.020	0.584	0.826
SOFA score	0.827	< 0.001	0.722	0.932
Combination of MO1/monocyte with SOFA	0.867	< 0.001	0.789	0.946

MO1 CD14 +  + CD16 − monocyte

*SOFA* Sequential organ failure assessment

### Value of MO1/monocytes for predicting 28-day mortality in all sepsis patients

**Fig. 6:**
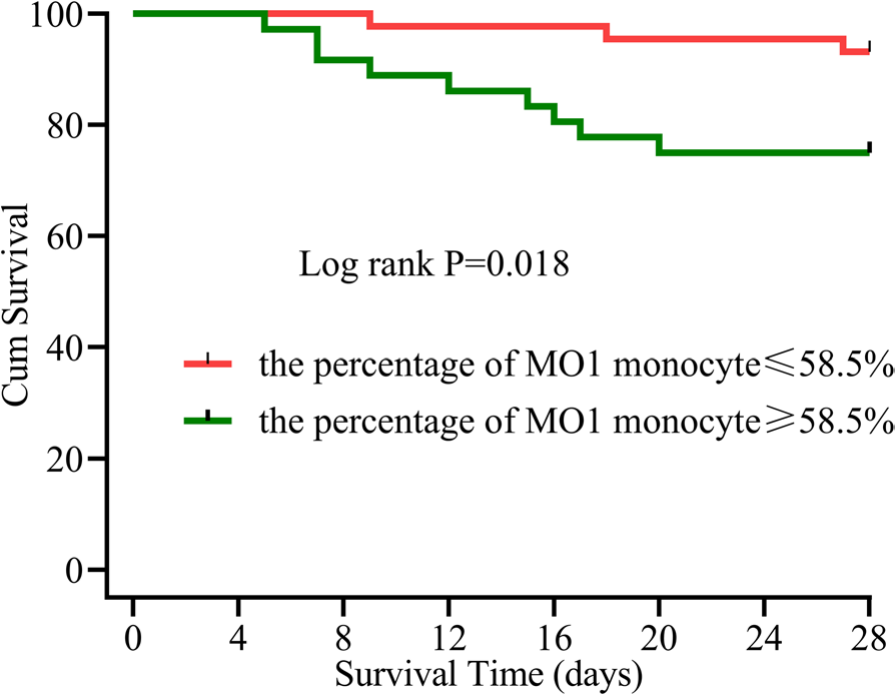
Survival curves of patients according to the percentage of MO1 monocyte

We further explored the significance of parameters in predicting 28-day mortality in all patients. The ROC curve showed that 58.5% was the optimal threshold in MO1/monocyte for predicting 28-day mortality in patients. The sensitivity, specificity, positive predictive value, and negative predictive value were 84.6%, 50.7%, 77.4%, and 62.2%, respectively. Using cut-off values determined by ROC curves, sepsis patients with a percentage of MO1/monocytes > 58.5% had a lower probability of survival on day 28 than patients with lower MO1/monocyte percentages (Fig. 6).

## Discussion

Aging is associated with impaired immune function that leads to older adult becoming less responsive to myriad pathogen and more susceptible to a series of infections ultimately. Changes in cellular phenotypes and functions in immune cells with aging have been found. Our study demonstrated that the percentage of MO3 monocytes (CD14 + CD16 + +) was higher in all Chinese Han older adult sepsis patients than in controls. This is consistent with previous report that the proportion of monocyte subsets appears to an expansion of non‐classical (CD14 + CD16 + +) monocytes in older adult populations [7]. The CD14 + CD16 +  + monocytes are also inflammatory cells owing to their potent pro-inflammatory activity [8]. *In vitro*, CD14 + CD16 +  + monocytes produce higher amounts of the pro-inflammatory factor TNF-α and lower amounts of the anti-inflammatory cytokine IL-10 in response to Toll-like receptor stimulation [9, 10]. *In vivo*, the CD14 + CD16 +  + monocyte population expands during infections, especially in sepsis [11]. All *in vitro* and *in vivo* studies have verified the clinical significance of CD14 + CD16 +  + monocytes during inflammation. However, CD14 + CD16 +  + monocytes also have a reduced phagocytic capacity by expressing lower levels of CCR2 (a chemokine receptor mediating monocyte chemotaxis during inflammation), and higher levels of CX3CR1 (a chemokine receptor mediating resident monocyte accumulation) [12], which implies that CD14 + CD16 +  + monocytes also have an anti-inflammatory function by reducing phagocytic capacity [12]. This confusion regarding the characterization of human monocyte subsets may be due to the different immune statuses in various age groups. Aging increases the proportion of CD14 + CD16 +  + monocytes in the circulation [13]. Studies on aging and monocytes have also shown that CD14 + CD16 +  + monocytes exhibit various features of cellular senescence [14], and senescent cells accumulate with aging [15]. These cells typically undergo extensive changes in protein expression and secretion, resulting in the persistent secretion of pro-inflammatory cytokines [16]. The CD14 + CD16 +  + monocytes also display reduced mitochondrial function in aging populations, which may enhance their reliance on pro-inflammatory glycolysis for ATP production [17], indicating a potential association between aging and changes in monocyte subset proportions and function. Our finding of the increased MO3 (CD14 + CD16 + +)/monocyte percentage in older adult Chinese Han sepsis verified the role of CD14 + CD16 +  + monocytes in the occurrence of this disease.

Classical monocytes (CD14 +  + CD16 −) are prominent monocytes in healthy individuals [18]. Substantially more evidence supports that CD14 +  + CD16 − monocytes are pro-inflammatory cells due to their high abilities of secreting pro-inflammatory cytokines in response to microbial products [6]. In a neonatal population, sepsis patients exhibited a significant increase in CD14 +  + CD16 − monocytes compared with controls, and CD14 +  + CD16 − monocytes demonstrated better diagnostic and prognostic abilities in ROC analysis [19]. One research about Gram-negative sepsis show that the absolute counts of CD14 +  + CD16 − monocytes on day 1 are higher in survivors compared than in non-survivors [20]. However, our study demonstrated that the percentage of MO1/monocytes (CD14 +  + CD16 −) in survival group was lower than death group according to the 28-day mortality. Moreover, CD14 +  + CD16 − monocytes were associated with a worse disease severity and prognosis in a subsequent analysis. This contradicting finding with the previous study may be explained by aging. Because research show that the proportion of monocyte subsets appears reducing in classical monocyte (CD14 + CD16 −) in elderly individuals [7]. Monocytes from older adults exhibit increased cytokine production compared with those from younger adults [21]. Transcriptomic profiling studies suggest that the proliferative capacity of CD14 +  + CD16 − monocytes may decline with age [22], and the proportion of CD14 +  + CD16 − monocytes in older adults is reduced compared with that in younger adults [13]. A recent review described that glycolysis contributes to increased inflammation, while slower fatty acid oxidation contribute to anti-inflammatory activities [23]. Fatty acid oxidation occurs in the mitochondria and aging impairs mitochondrial respiration in CD14 +  + CD16 − monocytes [13]. Thus, mitochondrial dysfunction could suppress anti-inflammatory cellular activities, enhancing inflammation. Hence, the increasing MO1/monocyte (CD14 +  + CD16 −) percentage in septic shock and 28-day survival patients in our study revealed early excessive inflammatory response in older adult patients with sepsis is an important underlying factor contributing to its severity and poor prognosis.

Human aging is associated with changes in the inflammatory cytokines IL-8 and MCP-1 synthesized by monocytes [24]. The release of a platelet granule protein causes the translocation of NF-κb into the nucleus of monocytes and triggers the synthesis of IL-8 and MCP-1 in older adult [25]. Increased levels of IL-6, IL-8, and MCP-1 during aging may contribute to adverse outcomes in older adult [26–28]. Our findings of upregulated IL-6, IL-8, and MCP-1 plasma levels according to 28-day mortality reconfirmed the previous results that excess inflammatory cytokines indicate poor prognosis. When monocytes are inactivated, they show a reduced ability to release pro-inflammatory cytokines, such as TNF-α, IL-1β, IL-6, and IL-12, and an increased capacity to secrete anti-inflammatory mediators, such as IL-10, as the disease progresses [29]. Our research showed that plasma IL-10 levels were significantly increased in all patients with sepsis, especially in the septic shock and 28-day mortality groups. This may indicate that an imbalance in cytokine release at the beginning of sepsis is a true state in older adult patients with sepsis. Among the four differentially expressed cytokines, the plasma MCP-1 level showed superior predictive value for the occurrence of sepsis, both alone and in combination with monocyte subtypes. This is consistent with the previous results that increased MCP-1 levels are associated with the highest mortality at 30 days and 6 months compared with lower levels in sepsis patients [30]. Classical monocytes secrete high levels of IL-8, IL-10, and MCP-1 *in vitro* [6]. Monocytes from older adult people secrete more IL-8 and MCP-1 than those from younger people in the presence of autologous platelets [24], indicating that the ability of monocytes to synthesize cytokines is altered with aging and disease.

The immune status of people of different ages is different and can be reflected by the changes in the proportion of peripheral blood monocyte subsets and their ability to secrete cytokines. The peripheral blood monocyte subsets combined with their cytokine expression provide a new predictor for early diagnosis, disease severity and prognosis in the older adult Chinese Han sepsis population.

## Limitations

Our study had several limitations. First, the results cannot fully reflect the real true nature due to the limited number of samples in this study. Therefore, the sample size should be enlarged in future research. Second, our study focused on monocyte subsets and cytokine secretion at the beginning of sepsis, but these states may change along with the course of sepsis. Therefore, dynamic analysis in monocyte subsets and cytokine secretion in different stages of sepsis require further research.

## Conclusions

This is the first study on the association of monocyte subsets and cytokine secretion with the occurrence and prognosis of sepsis in the older adult Chinese Han population aged > 65 years. Population aging is becoming increasingly serious worldwide, older adult sepsis is increasing. Therefore, it is particularly important to find new and effective indicators for early diagnosis and judgment of severity of sepsis. The conclusions of this study provide a basis for further studies on the immune status of older adult sepsis.

## Data Availability

The data that support the findings of this study are available from ResMan (http://www.medresman.org.cn/login.aspx) but restrictions apply to the availability of these data, which were used under license for the current study, and so are not publicly available. Data are however available from the authors upon reasonable request and with permission of ResMan.

## Abbreviations

ROC: Receiver operating characteristic
AUC: Under the curve
SOFA: Sequential organ failure assessment
ED: Emergency department
EDTA: Ethylenediaminetetraacetic acid
IFN: Interferon
MCP: Monocyte chemoattractant protein
TNF: Tumor necrosis factor
SASP II: Simplified acute physiology II
